# Identification and Characterization of Glycine‐ and Proline‐Rich Antioxidant Peptides From Antler Residues Based on Peptidomics, Machine Learning, and Molecular Docking

**DOI:** 10.1002/fsn3.70878

**Published:** 2025-09-01

**Authors:** Xingyu Xiao, Xi Chen, Libo Zhang, Yi Li, Qinchuan Lv, Tong Su, Jiayuan Fang, Shuo Zheng, Xunming Zhang, Linlin Hao, Shuqin Cheng

**Affiliations:** ^1^ College of Animal Science Jilin University Changchun China; ^2^ College of Veterinary Medicine Jilin University Changchun China; ^3^ Handan Vocational College of Science and Technology Handan China

**Keywords:** antioxidant peptides, antler residues, Keap1, machine learning, molecular docking, peptidomics

## Abstract

The purpose of this study was to determine the optimal enzymatic hydrolysis process for antler residues and to identify and characterize the antioxidant peptides present in the products of this hydrolysis. The results indicated that when neutrase is employed as the hydrolase, with an enzyme concentration of 2300 U/g, a temperature of 45°C, a pH of 6.0, a hydrolysis duration of 7.5 h, and a water/material ratio of 15%, the antler residual peptide (ARP) exhibited the highest DPPH clearance rate and degree of hydrolysis. Characterization of the physical and chemical properties revealed that ARP is a mixed antioxidant peptide rich in glycine and proline, with 93.6% of its composition consisting of peptides with a molecular weight of lower than 1 kDa. Furthermore, 21 antioxidant peptides were successfully identified from ARP using peptidomics and machine learning techniques. FM7, GPG10, and PP9 demonstrated advantages such as non‐toxicity, solubility in water, cell penetration, and the absence of digestive enzyme action sites. Additionally, molecular docking results showed that the binding energies of FM7, GPG10, and PP9 with Keap1 were 9.5, 8.5, and 10.8 kcal/mol, respectively, suggesting that these three antioxidant peptides may exert their antioxidant effects through the Nrf2/Keap1 pathway.

## Graphical abstract

ARP is a mixed antioxidant peptide rich in glycine and proline. Twenty‐one antioxidant peptides were identified through peptidomics and machine learning. Molecular docking predicted 21 antioxidant peptides can interact with DPPH/ABTS.

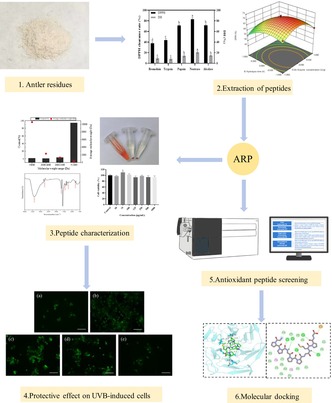

## 1 Introduction

Bioactive peptides are functional short peptides composed of 3–20 amino acids that exhibit specific biological activities (Chen et al. 2019; Zhang et al. 2019). Based on their functions, bioactive peptides can be categorized into several types, including antioxidant peptides, antibacterial peptides, antihypertensive peptides, and antitumor peptides (Li et al. 2019; Pellegrino et al. 2022). Among these, antioxidant peptides are among the most extensively studied (Tkaczewska et al. 2019). Antioxidant peptides can directly scavenge free radicals, chelate metal ions such as Fe^2+^ and Cu^2+^, and activate antioxidant enzymes, including superoxide dismutase (SOD), catalase (CAT), and glutathione peroxidase (GSH‐Px) (Liu et al. 2023; Ospina‐Quiroga et al. 2022). By exerting antioxidant effects, they help mitigate damage caused by oxidative stress, which arises from an imbalance between oxidation and antioxidant systems in the body (Sanford et al. 2020). Consequently, antioxidant peptides serve as viable alternatives to synthetic antioxidants and find widespread applications in food, medicine, cosmetics, and other industries.

Deer antlers are the regenerating bone appendages of deer, rich in bioactive compounds with various pharmacological functions (Dolan et al. 2022; Hong et al. 2021). Our previous research indicates that collagen derived from ossified deer antler tissue exhibits exceptionally high antioxidant activity following hydrolysis (Chen et al. 2023). The by‐product of this process, known as antler residue, contains significant amounts of underutilized protein. These residues are often discarded as waste, representing a considerable loss of resources. Consequently, further enzymatic hydrolysis of antler residues could enhance the high‐value utilization of antler and improve the economic benefits of the deer breeding industry.

The application of omics plays a crucial role in research within the life sciences (Nishiumi et al. 2014). As an emerging tool derived from proteomics, peptidomics enables the identification of peptide sequences in samples without the need for fractionation and separation (Zhang et al. 2020; Zheng et al. 2020). This approach eliminates cumbersome separation and purification steps, facilitating protein digestion and characterization of drugs, as well as the identification of disease peptide biomarkers, among other applications (Parada et al. 2022; Secher et al. 2016; Wu et al. 2019). Based on machine learning, dozens to tens of thousands of peptide sequences identified by peptidomics can be batch screened for whether and what kind of biological activity they have. Consequently, the integration of peptidomics with machine learning can significantly reduce the screening cycle for bioactive peptides, lower costs, and enhance screening accuracy. For instance, Chunxin Wang et al. (2024) screened umami peptides present in fermented seabass using peptidomics and machine learning, while Yu et al. (2024) employed these technologies to identify and predict bitter peptides from milk. Furthermore, molecular docking, a theoretical research method, is frequently utilized to investigate the binding modes and affinities of the screened bioactive peptides with their targets (Zhang et al. 2023; Zhao et al. 2023; Zhu, Xiong, et al. 2022). For example, (Fan, Han, et al. 2023) further investigated the antioxidant activity of peptides derived from duck liver by docking the identified antioxidant peptides with DPPH and ABTS, while Lee et al. (Lee et al. 2024) analyzed the potential antioxidant and anti‐inflammatory mechanisms of peptides through docking with Keap1 and IKK‐β.

In this study, single‐factor experiments and response surface methodology were initially employed to determine the optimal process for extracting antler residues peptides (ARP), focusing on maximizing antioxidant activity and hydrolysis degree. Subsequently, the protective effects and mechanisms of the extracted ARP against oxidative damage were investigated using a UVB‐induced HaCaT cell model. Additionally, a peptidomics approach combined with machine learning was utilized to identify and screen the antioxidant peptides present in ARP. Finally, the identified antioxidant peptides were docked with DPPH, ABTS, and Keap1 to explore their antioxidant action sites and mechanisms. This research holds significant implications for enhancing the economic value of the deer breeding industry and expanding the sources of antioxidant peptides.

## 2 Material and Methods

### 2.1 Materials and Chemical Reagents

Antler residue was purchased from Chengsheng Deer Industry Co. Ltd. (Changchun, China). Neutrase (100 U/mg), alcalase (200 U/mg), papain (800 U/mg), bromelain (600 U/mg), and trypsin (250 U/mg) were obtained from Yuanye Biotechnology (Shanghai, China). l‐Glutathione (GSH) was from Solarbio SCI & TECH Co. Ltd. (Beijing, China); 1,1‐Diphenyl‐2‐picrylhydrazyl (DPPH) was purchased from Tokyo Chemical Industry Co. Ltd. (Tokyo, Japan). DMEM, penicillin–streptomycin, trypsin, 2,2′‐Azino‐bis (3‐ethylbenzothiazoline‐6‐sulfonic acid) (ABTS), Nitrotetrazolium Blue chloride (NBT), Nicotinamide adenine dinucleotide (NADH) and Phenazine methosulfate (PMS) were purchased from Sigma‐Aldrich (St. Louis, MO, USA). Fetal bovine serum (FBS) was obtained from Gibco (Grand Island, NY, USA). Reactive oxygen species (ROS) assay kits were purchased from Beyotime Biotechnology (Shanghai, China). CAT, SOD, GSH‐Px and malondialdehyde (MDA) assay kits were obtained from Nanjing Jiancheng Bioengineering Institute (Nanjing, China). All other chemicals and solvents were of analytical grade. The HaCaT cell line utilized in this study was generously provided by Professor Hongsheng Ouyang and is currently stored in our laboratory.

### 2.2 Selection of Hydrolytic Enzymes

Disperse the antler residues in distilled water at a ratio of 1:10 (W/V) and mix well. Next, add five enzymes (neutrase, alcalase, papain, bromelain, and trypsin) with a concentration of 2500 U/g to each enzyme. Allow hydrolysis to occur for 4 h under optimal pH and temperature conditions. Following this, heat the mixture to 90°C for 15 min to deactivate the enzymes. Centrifuge the hydrolyzate at 8000 rpm (4°C) for 20 min, discard the precipitate, and collect the ARP, which is a combination of peptides. Finally, freeze‐dry the ARP and store it at −20°C.

### 2.3 Single Factor Experiment

The single‐factor experiment investigated the hydrolysis conditions, including enzyme concentration (1000, 2000, 3000, 4000, and 5000 U/g), temperature (35°C, 40°C, 45°C, 50°C, and 55°C), pH value (6, 6.5, 7, 7.5, and 8), water/material ratio (5%, 10%, 15%, 20%, and 25%), and hydrolysis time (7, 8, 9, and 10 h). These factors were studied for their effects on DPPH free radical scavenging activity.

### 2.4 Optimization by Response Surface Methodology (RSM)

The study utilized the RSM method to investigate the impact of various hydrolysis conditions on DPPH scavenging activity. Enzyme concentration (A), hydrolysis time (B), and pH value (C) were chosen as independent variables based on single‐factor experiments, with the DPPH free radical scavenging rate as the response value. Design Expert was employed to plan the experiment and predict the DPPH value. By conducting multi‐factor variance analysis and utilizing a second‐order model for prediction, the optimal enzymatic hydrolysis conditions were determined through the response surface model. The experimental factors and their level settings are detailed in Table S1.

### 2.5 Molecular Weight (Mw) Distribution

A 10 mL volumetric flask was filled with 100 mg of ARP and then diluted with 10 mL of deionized water. The solution was sonicated for 5 min and centrifuged at 10,000 rpm for 5 min. The resulting supernatant was filtered through a 0.22 μm membrane, and the molecular weight distribution of ARP was analyzed using high‐performance liquid chromatography (HPLC). The mobile phase consisted of acetonitrile/water/trifluoroacetic acid in a 40/60/0.1 (v/v) ratio, with detection at 220 nm, an injection volume of 10 μL, and a flow rate of 0.5 mL/min. A linear standard curve of log MW versus retention time was established using cytochrome C (12,384 Da), bacitracin (1422 Da), Gly–Gly–Try–Arg (451 Da), and Gly–Gly–Gly (12,384 Da).

### 2.6 Amino Acid Composition Analysis

Place ARP into a hydrolysis tube, add 10 mL of 6 mol/L HCl, and position it in an oil bath at 110°C for hydrolysis over a period of 24 h. After the hydrolysis is complete, allow the mixture to cool to room temperature, then transfer it to a rotary evaporator for deacidification. Once dried, add 2 mL of sodium citrate buffer to ensure complete dissolution, filter the solution through a 0.45 μm filter, and analyze the resulting solution using a Biochrom 30+ amino acid analyzer (Biochrom, Cambridge, UK).

### 2.7 UV Absorption and FTIR Assay

The UV spectrum of ARP powder was measured using a UV–Vis–NIR spectrophotometer (Shimadzu Corporation, Tokyo, Japan), scanning range 200–800 nm. ARP powder was mixed and ground with potassium bromide powder (1:100), followed by samples being analyzed using an IS5 FTIR spectrometer (Thermo Fisher, Waltham, MA, USA) with a scan range of 500–4000 cm^−1^.

### 2.8 Determination Antioxidant Activity and Degree of Hydrolysis

#### 2.8.1 Degree of Hydrolysis (DH)

Following a similar procedure as described previously (Shanggui et al. 2008), 5 mL of enzyme solution was titrated to pH 8.2 using 1 mol/L NaOH. Subsequently, 10.0 mL of neutral formaldehyde solution was added to the mixture, and the pH was adjusted to 9.2. The volume of NaOH required to increase the pH from 8.2 to 9.2 was noted as V_1_. A blank solution was prepared using deionized water instead of the enzymatic hydrolysis solution, and the volume of NaOH consumed was recorded as *V*_0_. The DH was calculated using the following equation:

$$\mathrm{DH}\;\left(\%\right)=\left[C\times \left(V_{1}-V_{0}\right)\times V\times 0.014/\left(5\times M\right)\right]\times 100\%$$

where *C* is the concentration of NaOH used for titration (mol/L), *V* is the total volume of hydrolysate, *M* is the total nitrogen of velvet antler residue (g/g).

#### 2.8.2 DPPH Radical Scavenging Activity

The DPPH radical scavenging activity of ARP was evaluated using a modified method (Fan, Ge, et al. 2023). A sample solution of 100 μL was combined with an equal volume of DPPH solution (200 μM in absolute ethanol). This mixture was then incubated in the dark at 25°C for 30 min, and the absorbance was measured at 517 nm using a microplate spectrophotometer (Biotek, USA). The rate of scavenging DPPH radicals was evaluated as follows:

$$\text{DPPH scavenging rate}\;\left(\%\right)=\left[1-\left(\mathrm{As}-\mathrm{Ab}\right)/\mathrm{Ac}\right]\times 100\%$$

where As and Ab are the absorbance of the sample and blank respectively, Ac is the absorbance of thecontrol.

#### 2.8.3 ABTS Radical Scavenging Capacity

The ABTS radical scavenging activities of the ARP were determined following a modified method (Wen et al. 2020). A sample solution (10 μL) was mixed with diluted ABTS solution (200 μL) and left in the dark for 6 min. The absorbance was measured at 734 nm. The ABTS scavenging rate was calculated using the formula:

$$\text{ABTS scavenging rate}\;\left(\%\right)=\left[1-\left(\mathrm{As}-\mathrm{Ab}\right)/\mathrm{Ac}\right]\times 100\%$$

where As is the absorbance of the sample, Ab is the absorbance of the blank, and Ac is the absorbance of the control.

#### 2.8.4 Superoxide Anion Scavenging Ability

The scavenging activity of ARP on superoxide anion was assessed using a modified method (Wang, Zhao, et al. 2020). A mixture of 1 mL of sample solution, 1 mL of NADH (2.52 mM), and 1 mL of NBT (0.12 mM) was prepared. Subsequently, 1 mL of PMS was added, and the absorbance at 560 nm was measured after incubating at 25°C for 5 min in the dark. Water was used as a substitute for the sample in the control group. The superoxide anion radical scavenging rate was calculated using the formula:

$$\text{Superoxide anion scavenging rate}\;\left(\%\right)=\left[1-\left(\mathrm{As}/\mathrm{Ac}\right)\right]\times 100\%$$

where As and Ac represent the absorbance of the sample and control, respectively.

#### 2.8.5 Hydroxyl Radical Scavenging Activity

The hydroxyl radical scavenging activity of ARP was assessed following a previously established method with minor adjustments (Cao et al. 2003). A sample of 100 μL was combined with an equal volume of FeSO_4_ (7.5 mM), hydrogen peroxide (0.03% [v/v]), and salicylic acid (7.5 mM), then incubated at 37°C for 30 min. Water served as the blank sample and control. The absorbance was recorded at 510 nm, and the hydroxyl radical scavenging rate was calculated using the formula:

$$\text{Hydroxyl radical scavenging rate}\;\left(\%\right)=\left[1-\left(\mathrm{As}-\mathrm{Ab}\right)/\mathrm{Ac}\right]\times 100\%$$

where As and Ab represent the absorbance of the sample and blank, respectively, and Ac is the absorbance of the control.

### 2.9 Cytotoxicity Assay and Hemolysis Test

The crude extract of the enzymatic hydrolysate ARP was dissolved in DMEM medium to create DMEM medium with varying concentrations of ARP (50, 75, 100, 125, 250, 500, and 1000 μg/mL). Following the cultivation of HaCaT cells (1 × 10^4^ cells/well) in 96‐well plates for 24 h, the medium was aspirated and discarded. The experimental groups received DMEM medium containing different concentrations of ARP, while the control group was provided with an equivalent volume of DMEM medium devoid of ARP. After an additional 24‐h incubation, absorbance was measured at 450 nm using the CCK‐8 method, and cell viability was calculated using the following formula:

$$\text{Cell viability}\;\left(\%\right)=\mathrm{As}/\mathrm{Ac}\times 100\%$$

where As is the sample absorbance and Ac is the control absorbance.

The specific experimental steps of the hemolysis test conducted in accordance with the method described by Pimchan et al. (2023), with modifications. In brief, collected mouse blood cells were washed three times with PBS and subsequently resuspended in PBS to achieve a final concentration of 1% (v/v) red blood cells. Then 50 μL of the red blood cell suspension was incubated with 50 μL of ARP (1, 2, 4, 8, 12, and 16 mg/mL) at 37°C for 1 h, after which the absorbance was measured at 570 nm. PBS and 1% Triton‐X‐100 served as positive and negative controls, respectively, in this experiment.

### 2.10 Effects of ARP on the UVB‐Induced HaCaT Cells

HaCaT cells were seeded in 96‐well plates at a density of 1 × 10^4^ and cultured for 24 h. Subsequently, the cells were divided into experimental and control groups. The control group was irradiated with UVB at varying doses (10, 20, 30, 40, 50, and 60 mJ/cm^2^) to determine the radiation dose corresponding to half of the inhibition of cell viability. To assess the protective effect of ARP on UVB‐induced HaCaT cells, medium containing different concentrations of ARP (0, 50, 75, 100, 125, and 250 μg/mL) was used to treat the cells for 30 min. Subsequently, the cells were washed with PBS and irradiated with a radiation dose corresponding to half suppression. Following the radiation, the PBS was replaced with medium, and then the cells were incubated with CCK‐8 solution. The absorbance was then measured at 450 nm using a microplate reader.

### 2.11 Detection of Antioxidant Indicators in UVB‐Induced HaCaT Cells

HaCaT cells were cultured in 6‐well plates (3 × 10^5^ cells/well) at 37°C for 24 h, and then ARP (50, 75, and 100 μg/mL) was added to the experimental group and incubated for 24 h. After treatment, both the experimental group and the control group received 40 mJ/cm^2^ UVB irradiation. Afterwards, assay kits were used to evaluate the activities of CAT and SOD, as well as the MDA levels.

### 2.12 Determination of ROS Levels in UVB‐Induced HaCaT Cells

Following incubation with ARP at concentrations of 50, 75, and 100 μg/mL for 24 h, the cells (3 × 10^5^ cells/well) were exposed to 40 mJ/cm^2^ UVB radiation. Subsequently, the cells were treated with 10 μM DCFH‐DA for 20 min at 37°C and then rinsed with DMEM. For fluorescence microscopic analysis, PBS was substituted for DMEM, and the relative fluorescence intensity was quantified using Image J software. The level of ROS was assessed using a specific kit.

### 2.13 Identification of Peptide Sequences by LC–MS/MS

ARP was characterized using LC–MS/MS to determine the peptide sequences it contained. The instrument employed was the VANQUISH NEO nanoliter liquid chromatography system (Thermo Scientific), with the column temperature maintained at 55°C. Mobile phase A comprised 0.1% formic acid in water, while mobile phase B consisted of 80% acetonitrile containing 0.1% formic acid. The gradient elution program is as follows: 0–1.8 min, 4.0%–4.5% B, 0.50 μL/min; 1.8–2.0 min, 4.5%–5.0% B, 0.50 μL/min; 2.0–41.0 min, 5.0%–20.0% B, 0.30 μL/min; 41.0–57.0 min, 20.0%–35.0% B, 0.30 μL/min; 57.0–57.5 min, 35.0%–55.0% B, 0.30 μL/min; 57.0–58.0 min, 55.0%–99.0% B, 0.50 μL/min; 58.0–60.0 min, 99.0% B, 0.50 μL/min. The MS/MS original files were analyzed using Proteome Discoverer software (version 3.0). Subsequently, a FASTA database containing Cervidae protein sequences was constructed and searched to obtain peptidomics information regarding ARP.

### 2.14 Machine Learning Screening of Antioxidant Peptides

The PeptideRanker database (http://distilldeep.ucd.ie/PeptideRanker/) was utilized to predict the probability of biological activity for the obtained peptide sequences. A higher PeptideRanker score indicates a greater likelihood that the peptide is biologically active. Studies suggest that when the PeptideRanker score exceeds 0.5 (Zhu, Zhu, et al. 2022), the peptide sequence can be regarded as biologically active (Iwaniak et al. 2020). To enhance the likelihood of identifying bioactive peptides, the threshold for the PeptideRanker score is set at 0.95. Consequently, peptides with scores greater than 0.95 are selected for further evaluation of their antioxidant activity using the BIOPEP database (https://biochemia.uwm.edu.pl/biopep/start_biopep.php).

### 2.15 Antioxidant Peptide Property Prediction

Utilize APD3 (https://aps.unmc.edu/AP/) to predict the Mw and grand average of hydropathy values (GRAVY) of the peptide. Employ ToxinPred (https://webs.iiitd.edu.in/raghava/toxinpred/) to assess the peptide's toxicity. The potential for cell penetration of the peptides can be evaluated using CPPpred (http://distilldeep.ucd.ie/CPPpred/). Additionally, use the online tool available (https://www.novopro.cn/tools/protease‐digestion‐tool.html) to determine the presence of trypsin/pepsin/trypsin digestive sites within the peptide sequence. Furthermore, analyze the amino acid composition of the screened peptides.

### 2.16 Molecular Docking

The 21 antioxidant peptides screened were molecularly docked with DPPH and ABTS to assess their antioxidant activity. The structures of the DPPH radical (CID: 2735032) and ABTS radical (CID: 5360881) were obtained from the PubChem database (Zhang et al. 2025). Prior to docking, peptide modeling was conducted using the online platform (https://cloud.yinfotek.com). The DPPH, ABTS, and peptide files were imported in PDB format into AutoDockTools (v1.5.7), where settings such as water removal, hydrogenation, charge calculations, and bond rotation were performed, followed by saving the files in “pdbqt” format. AutoDock Vina software was employed for docking (Trott and Olson 2010), utilizing DPPH and ABTS as ligands and the peptides as receptors. The docking results were subsequently analyzed using PyMOL 2.3.0 and Discovery Studio 2019.

The final antioxidant peptide was selected for docking with Keap1 to investigate its potential antioxidant effects via the Nrf2/Keap1 pathway. The crystal structure of Keap1 (PDB ID: 2FLU) was obtained from the Protein Data Bank (PDB, https://www.rcsb.org/). The preparation of Keap1 involved removing water, deleting ligands, adding hydrogen, calculating charges, and subsequently saving the structure as a pdbqt file. The calculated binding sites of Keap1 were identified as *x*: 5, *y*: 9, *z*: 2, which align closely with the binding site determinations made by Wei et al. (2022). Docking and analysis were conducted following the same procedural steps.

### 2.17 Statistical Analysis

Each experiment was conducted a minimum of three times. The experimental results are presented as the mean ± standard deviation and were analyzed between groups using one‐way analysis of variance (ANOVA) with GraphPad Prism 9.0 (San Diego, USA). Statistically significant differences are indicated by *p < 0.05, **p < 0.01, ***p < 0.001, or ^#^ *p* < 0.05, ^##^ *p* < 0.01, ^###^ *p* < 0.001.

## 3 Result and Discussion

### 3.1 Selection of Optimal Hydrolase for APR

**FIGURE 1:**
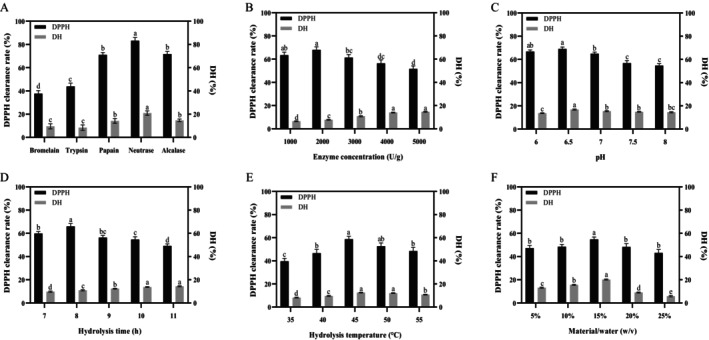
Protease selection and enzymatic hydrolysis single factor optimization (A–F). (A) Protease selection; (B) enzyme concentration; (C) pH; (D) hydrolysis time; (E) hydrolysis temperature; (F) water/material ratio. All results were triplicates of the mean ± SD. Different lowercase letters (a–d) represent significant differences (*p* < 0.05).

Figure 1A illustrates the DPPH scavenging efficiency and degree of hydrolysis of enzymatic products obtained from antler residues treated with different proteases. The results revealed variations in DPPH radical scavenging efficiency and degree of hydrolysis among the enzymatic products from the five proteases. Notably, the enzymatic product from neutrase exhibited superior performance, with a DPPH scavenging rate of 83.20% and a DH of 20.95%. Consequently, this study identifies neutrase as the optimal hydrolyzing enzyme for future experiments.

### 3.2 Results of Single Factor Optimization

The enzymatic hydrolysis of velvet antler residues was conducted using neutrase, with DPPH clearance rate and DH as detection indicators. Five extraction conditions, including enzymatic hydrolysis time, temperature, pH, solid–liquid ratio, and enzyme concentration, were optimized according to Section 2.3. As illustrated in Figure 1B–F, during the optimization process, the DPPH clearance rate initially increased before subsequently decreasing under all five conditions. Therefore, the conditions corresponding to the peak DPPH clearance rate during the optimization process were selected as the optimal enzymatic hydrolysis conditions: an enzyme concentration of 2000 U/g, a temperature of 45°C, a pH of 6.5, an enzymatic hydrolysis time of 8 h, and a water/material ratio of 15%.

DH is a key metric for assessing the extent of protein hydrolysis and serves as a supplementary indicator for optimizing hydrolysis conditions (Amado et al. 2014). In the optimization of pH, hydrolysis temperature, and water/material ratio, the conditions corresponding to the highest DH coincided with those yielding the highest DPPH clearance rate. Conversely, in the optimization of enzyme concentration and hydrolysis time, the degree of DH peaked at an enzyme concentration of 4000 U/g and a hydrolysis time of 10 h; however, the corresponding DPPH clearance rate significantly decreased. Based on a comprehensive evaluation of DPPH clearance rate and DH, the optimal enzymatic hydrolysis conditions for ARP were determined to be: enzyme concentration of 2000 U/g, temperature of 45°C, pH of 6.5, enzymatic hydrolysis time of 8 h, and water/material ratio of 15%.

### 3.3 Response Surface Methodology for ARP Extraction

Following the outcomes of the single‐factor experiments, further optimization of enzyme dosage, enzyme digestion time, and pH was conducted using response surface methodology. A Box–Behnken design experiment was carried out with three factors at three levels, totaling 17 sets of tests, including five sets of center point experiments for error estimation. The experimental design and results for DPPH and DH are outlined in Table S2. Regression analysis of the data shows the relationship between DPPH clearance and DH in relation to enzyme concentration (A), enzyme digestion time (B), and pH (C).

$$\begin{array}{cc} \text{DPPH}\;\left(\%\right)= & 70.46-1.45A-4.14B-3.79C+0.1850\mathrm{AB}\\ & -0.5000\mathrm{BC}-4.72\mathrm{BC}-7.77A^{2}-4.92B^{2}-5.97C^{2} \end{array}$$

$$\begin{array}{cc} \mathrm{DH}\;\left(\%\right)= & 15.76+0.2050A-0.7962B+0.2187C-0.4150\mathrm{AB}\\ & -0.12\mathrm{AC}+0.3725\mathrm{BC}-2.57\;A^{2}-2.64B^{2}-2.31C^{2} \end{array}$$

Tables 1 and 2 display the *p*‐values for both the DPPH and DH models, which are below 0.001, demonstrating high significance. The non‐fit terms in the models have *p*‐values of 0.9560 and 21.56, respectively, exceeding 0.05, suggesting the absence of significant non‐fit errors and the accurate reflection of experimental data. The coefficients of determination (*R* ^2^) for the quadratic regression models are 0.9573 and 0.9766, indicating that they can explain 95.73% and 97.66% of the data variation, showing high data reliability. Furthermore, the corrected coefficients of determination ($R_{\mathrm{adj}}^{2}$) were 0.9023 and 0.9464, closely aligned with the corresponding *R* ^2^ values, signifying that the model predictions closely match the experimental outcomes, confirming the validity and reliability of the results.

**TABLE 1:** Regression equation analysis of variance (DPPH clearance rate).

Variables	Sum of squares	df	Mean square	*F*	*p*
Model	922.28	9	102.48	17.42	0.0005
A	16.82	1	16.82	2.86	0.1347
B	137.12	1	137.12	23.31	0.0019
C	114.91	1	114.91	19.54	0.0031
AB	0.1369	1	0.1369	0.0233	0.8831
AC	1.0000	1	1.0000	0.1700	0.6924
BC	89.11	1	89.11	15.15	0.0060
A^2^	254.43	1	254.43	43.25	0.0003
B^2^	101.86	1	101.86	17.32	0.0042
C^2^	150.24	1	150.24	25.54	0.0015
Residual	41.18	7	5.88		
Lack of fit	17.19	3	5.73	0.9560	0.4944
Pure error	23.98	4	6.00		
Cor total	963.46	16			
*R* ^2^ = 0.9573			$R_{\mathrm{adj}}^{2}$ = 0.9023		

**TABLE 2:** Regression equation variance analysis (degree of hydrolysis).

Variables	Sum of squares	df	Mean square	*F*	*p*
Model	100.08	9	11.12	32.40	< 0.0001
A	0.3362	1	0.3362	0.9795	0.3553
B	5.07	1	5.07	14.78	0.0063
C	0.3828	1	0.3828	1.12	0.3260
AB	0.6889	1	0.6889	2.02	0.1995
AC	4.16	1	4.16	12.13	0.0102
BC	0.5550	1	0.5550	1.62	0.2441
A^2^	27.83	1	27.83	81.07	< 0.0001
B^2^	29.31	1	29.31	85.39	< 0.0001
C^2^	22.43	1	22.43	65.36	< 0.0001
Residual	2.40	7	0.3432		
Lack of fit	2.26	3	0.7542	21.56	0.0062
Pure error	0.1399	4	0.0350		
Cor total	102.48	16			
*R* ^2^ = 0.9766			$R_{\mathrm{adj}}^{2}$ = 0.9464		

In the DPPH model, the *p*‐values of variables B, C, BC, A^2^, B^2^, and C^2^ were all below 0.01, signifying a significant or highly significant impact on the DPPH free radical scavenging rate. On the other hand, while the *p*‐value of variable A was higher, the *p*‐value of A^2^ was 0.0003, suggesting a nonlinear relationship with the DPPH free radical scavenging rate. In the DH model, the *p*‐values of A^2^, B^2^, and C^2^ were extremely low, below 0.0001, and the *p*‐values of B and AC were under 0.05, indicating a significant to highly significant influence on the hydrolysis degree. Despite the higher *p*‐values of A and C, the *p*‐values of their quadratic terms A^2^ and C^2^ were < 0.0001, revealing a non‐linear association with the hydrolysis degree.

**FIGURE 2:**
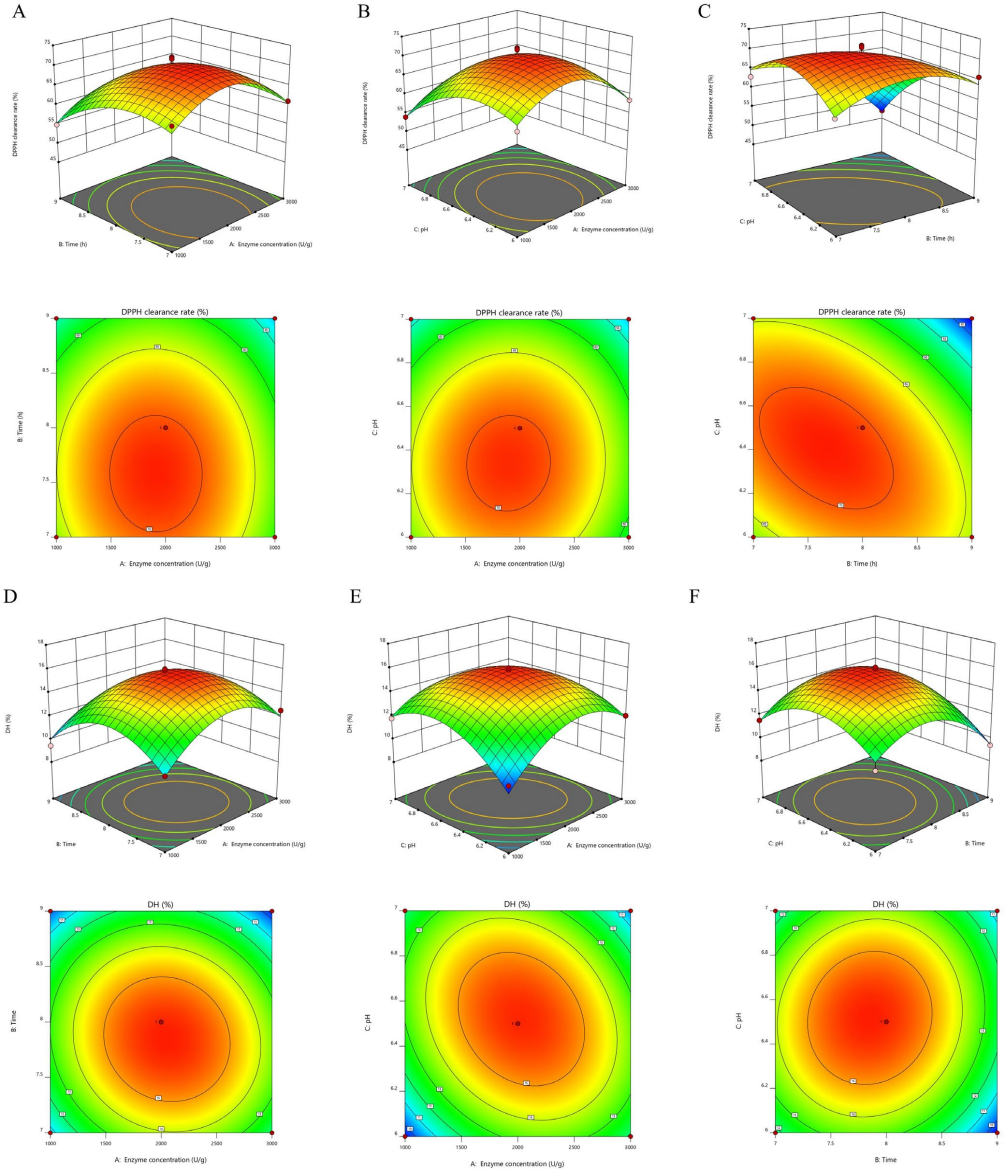
Response surface of DPPH clearance rate and DH for ARP. The upper images represent three‐dimensional response surface plots, while the lower images represent two‐dimensional contour plots. (A, D) Effect of enzyme concentration and hydrolysis time on the DPPH clearance rate and DH; (B, E) effect of enzyme concentration and pH on the DPPH clearance rate and DH; (C, F) effect of pH and hydrolysis time on the DPPH clearance rate and DH.

In addition, the utilization of 3D response surface and contour plots can provide a more intuitive representation of the strength of interactions between factors. We employed these plots for DPPH and DH to enhance our understanding of the interactions among various enzymatic factors (Figure 2).

The study utilized regression model analysis to determine the optimal conditions for the preparation of antioxidant peptides from antler residue. When using the DPPH radical scavenging rate as the evaluation criterion, the optimal conditions were found to be 2047.41 U/g of enzyme concentration, pH 6.07, and 7.57 h of hydrolysis time, resulting in an expected DPPH scavenging rate of 68.43%. On the other hand, when the degree of hydrolysis was used as the evaluation criterion, the optimal preparation conditions included 2558.8 U/g of enzyme concentration, pH 6.01, hydrolysis time of 7.42 h, and an expected degree of hydrolysis of 13.10%. To meet practical operational needs, the finalized process parameters were set at enzyme concentration of 2300 U/g, pH 6.0, and hydrolysis time of 7.5 h. This solution was validated through three replications, with measured DPPH removal rate of (68.26% ± 1.69%) and hydrolysis degree of (15.25% ± 0.43%), demonstrating consistency with the predicted values.

### 3.4 Molecular Weight Distribution of ARP

**FIGURE 3:**
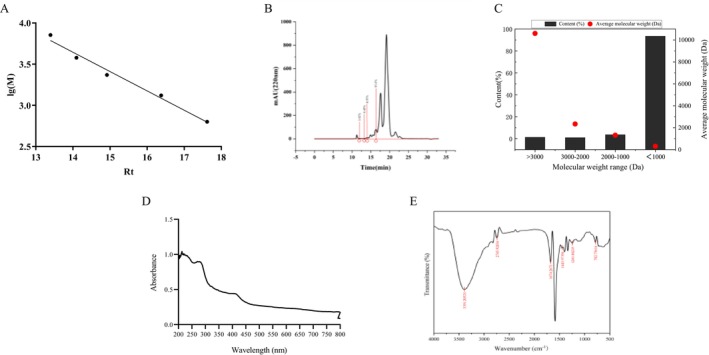
Characterization of ARP related properties. (A) Relative molecular mass standard curve of ARP; (B) elution peak of ARP; (C) molecular weight distribution of ARP; (D) UV absorption for ARP; (E) FTIR assay for ARP.

To illustrate the correlation between the logarithm of the relative Mw of ARP and elution time (Rt), an equation was derived with lg^(Mw)^ as the vertical axis and Rt as the horizontal axis: lg^(Mw)^ = −0.2349Rt + 6.934. The *R* ^2^ value for this equation was 0.9829, indicating a strong model fit that can accurately assess the distribution of Mw of ARP (Figure 3A). Multiple elution peaks were observed in ARP, suggesting that it comprises several fractions with varying relative molecular masses (Figure 3B). Both Figure 3C and Table S3 demonstrate that ARP is predominantly composed of peptides under 1 kDa, which account for as much as 93.6%, while only 0.83% of peptides exceed 10 kDa. This suggests that the antioxidant peptides in antler residue are predominantly low molecular weight peptides.

### 3.5 UV Absorption and FTIR Assay for ARP

Figure 3D illustrates the UV absorption characteristics of ARP within the wavelength range of 200–800 nm. A pronounced absorption peak is observed in the 200–220 nm region, where the absorbance reaches a notable maximum. This peak is primarily attributed to the π–π* electronic transition in the peptide bond (Qu et al. 2022; Theodoroula et al. 2022). Given the abundance of peptide bonds in the peptide, this electronic transition leads to substantial absorption in this wavelength range. The absorbance gradually diminishes as the wavelength increases, indicating the absence of other significant electronic transitions or chromophore absorption in this region, which aligns with the typical UV spectral characteristics of peptides.

Figure 3E presents vibrational information for the peptide in the wavenumber range of 500–4000 cm^−1^. A significant absorption peak is noted in the wavenumber range of 1600–1700 cm^−1^, corresponding to the amide I band, which arises from the stretching vibration of C=O in the peptide bond (Hu et al. 2022; Wang, Wang, and Wen 2020). This feature is a crucial indicator of the presence of a peptide structure. Additionally, the absorption peak observed near the wavenumber of 1500–1600 cm^−1^ pertains to the amide II band, generated by the coupling of N–H bending and C–N stretching vibrations (Chu et al. 2022). Furthermore, a broad absorption peak in the 3000–3500 cm^−1^ wavenumber region is primarily attributed to the stretching vibrations of N–H and O–H, suggesting the presence of hydrogen bonds within the peptide (Ren et al. 2022). These findings further confirm that ARP possesses a relatively intact peptide structure.

### 3.6 Amino Acid Composition of ARP

To enhance the understanding of the antioxidant properties of ARP, this study analyzed its amino acid composition (Table 3). The amino acid content in ARP is as high as 735.675 mg/g. Among these, the three most abundant amino acids are glycine (Gly), glutamic acid (Glu), and proline (Pro). Gly is present at a concentration of 174.087 mg/g, accounting for 23.66% of the total. Its simple structure and flexibility allow it to adjust the spatial conformation of the peptide chain (Shen and Ji 2015), making the antioxidant peptide more accessible to free radicals (Lu et al. 2022). The Glu is 91.807 mg/g, representing 12.48% of the total. Due to its carboxyl group, glutamic acid is highly hydrophilic. In an aqueous environment, this property not only aids in the dispersion of antioxidant peptides but also enables the carboxyl groups to participate in the electron transfer process (Pisarevsky et al. 2020; Wróbel et al. 2021). By engaging in redox reactions with free radicals, it effectively quenches active substances and mitigates the oxidation process (Wang et al. 2022). Pro is present at 89.197 mg/g, accounting for 12.12%. Its unique pyrrole ring structure allows it to donate protons through the pyrrole ring, facilitating the chelation of metal ions to scavenge free radicals (Wang et al. 2019). Additionally, basic amino acids (His, Lys, Arg) can provide hydrogen protons to electron‐deficient free radicals via positive charges or form chelates with oppositely charged metal ions, thereby neutralizing free radicals and preventing oxidative damage (Kong et al. 2019). These findings lay a solid foundation for further exploration of the antioxidant mechanisms of the studied ARP and its potential applications.

**TABLE 3:** Amino acid composition analysis results.

No.	Amino acids	Retention time (min)	Peak area (mVs)	Content (mg/g)	Proportion (%)
1	Asp	8.533	1927.475	53.244	7.24
2	Thr	10.321	834.643	19.814	2.69
3	Ser	11.213	1465.635	29.901	4.06
4	Glu	13.511	3096.582	91.807	12.48
5	Gly	19.427	14,402.39	174.087	23.66
6	Ala	20.519	3375.016	69.129	9.40
7	Cys	22.092	29.908	0.933	0.13
8	Val	22.653	943.556	21.458	2.92
9	Met	24.601	300.556	6.821	0.93
10	Ile	25.893	466.12	12.424	1.69
11	Leu	26.96	1263.462	28.284	3.8
12	Tyr	29.864	282.134	8.848	1.20
13	Phe	30.727	682.91	19.498	2.65
14	His	35.527	256.002	6.551	0.89
15	Lys	36.791	1451.458	36.948	5.02
16	Arg	45.313	2113.935	66.731	9.07
17	Pro	14.812	648.73	89.197	12.12
	Total	—	33,540.512	735.675	99.95

### 3.7 Free Radical Scavenging Ability for ARP

**FIGURE 4:**
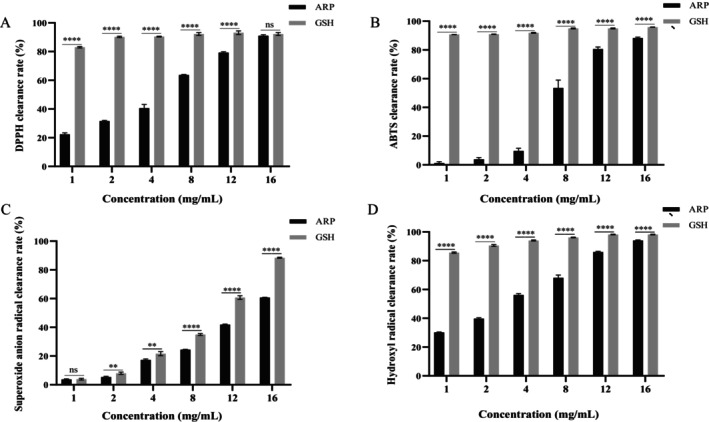
ARP scavenging ability of different free radicals. (A) DPPH free radical; (B) ABTS radical; (C) superoxide anion radical; (D) hydroxyl radical. Results were triplicates of the mean ± SD. **p < 0.01 and ****p < 0.0001 versus corresponding concentration of GSH.

To evaluate the antioxidant activity of ARP, its capacity to scavenge four distinct free radicals was assessed, using GSH as a control for comparison. The results are illustrated in Figure 4. ARP demonstrated dose‐dependent scavenging effects on four free radicals. Notably, ARP exhibited strong scavenging abilities for DPPH, ABTS free radicals, superoxide anion radicals, and hydroxyl radicals, with percentages of 91.01% ± 0.67%, 88.29% ± 0.59%, 60.73% ± 0.16%, and 94.11% ± 0.41%, respectively. These findings suggest that ARP possesses significant free radical scavenging activity and has the potential for application in anti‐aging health products and cosmetics.

### 3.8 Biosafety Assessment for ARP

**FIGURE 5:**
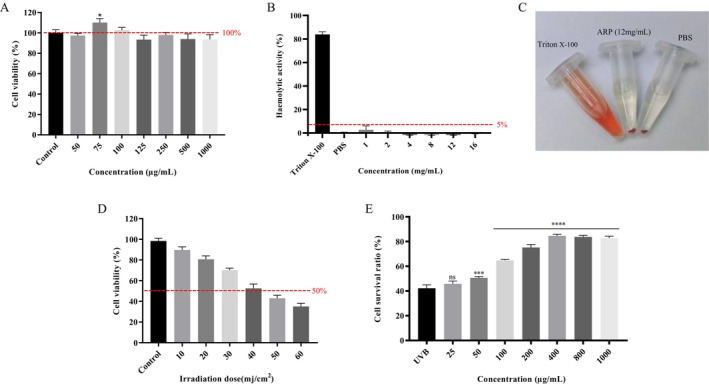
Biosafety assessment of ARP and its protective effect on UVB‐induced HaCaT cells. (A) Cytotoxicity of ARP. *p < 0.05 versus Control group; (B) hemolytic activity of ARP; (C) hemolytic activity physical picture for ARP; (D) UVB radiation dose selection; (E) protective effect of ARP on UVB‐induced HaCaT cells. Results were triplicates of the mean ± SD. ***p < 0.001 and ****p < 0.0001 versus UVB group.

The CCK‐8 experimental results (Figure 5A) demonstrate that the cell survival rate in the control group was nearly 100%, indicating normal cell growth in the absence of ARP treatment. As the ARP concentration increased from 50 to 1000 μg/mL, although the survival rates fluctuated, they remained above 90%. This suggests that ARP exhibits low toxicity or no toxicity to cells within this concentration range. In the hemolysis experiment, as illustrated in Figure 5B, the hemolytic activity of the ARP treatment group remained below 5% when the concentration increased from 1 to 12 mg/mL, indicating minimal damage to red blood cell membranes. Furthermore, Figure 5C shows that ARP does not cause hemolysis of red blood cells. Collectively, these results indicate that ARP possesses favorable biological safety as an antioxidant peptide.

### 3.9 Protective Effect of ARP on HaCaT Cells Damaged by UVB

HaCaT cells were irradiated with varying doses of UVB radiation, and the results indicated that cell viability gradually decreased as the UVB dose increased (Figure 5D). Specifically, at a UVB radiation dose of 40 mJ/cm^2^, cell viability was reduced by half. Consequently, a UVB dose of 40 mJ/cm^2^ was employed for subsequent experiments. Figure 5E illustrates that, under UVB irradiation, the cell activity of HaCaT increases with rising concentrations of ARP. This suggests that ARP exerts a protective effect on UVB‐induced HaCaT cells.

### 3.10 Protective Effects of ARP With UVB‐Induced Damage on HaCaT Cells

**FIGURE 6:**
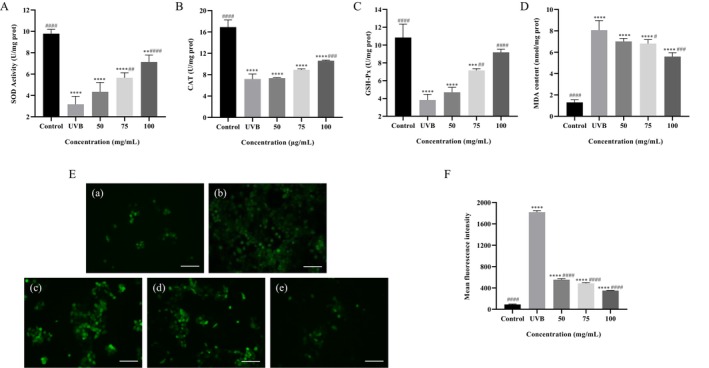
Effect of ARP on antioxidant enzymes, MDA, and ROS in UVB‐induced HaCaT cells. (A) SOD; (B) CAT; (C) GSH‐Px; (D) MDA; (E) ROS content under different concentrations of ARP; (F) mean fluorescence intensity of cells for ROS in different groups. Results were triplicates of the mean ± SD. **p < 0.01, ***p < 0.001and ****p < 0.0001 versus control group; ^#^ *p* < 0.05, ^##^ *p* < 0.01, ^###^ *p* < 0.001 and ^####^ *p* < 0.0001 versus UVB model group.

UVB radiation is a common model of cellular injury that directly disrupts biological membranes and induces lipid peroxidation. SOD, CAT, and GSH‐Px are key components of the intracellular antioxidant defense system, crucial for maintaining oxidative balance in the body (Rinnerthaler et al. 2015; Towbin et al. 1979). The results depicted in Figure 6A–D show that UVB exposure significantly decreased the activities of these antioxidant enzymes, indicating a notable impact of oxidative stress on the antioxidant system. However, when ARP was introduced to UVB‐damaged cells, the activities of SOD, CAT, and GSH‐Px increased in a dose‐dependent manner, surpassing those of cells treated with UVB alone (*p* < 0.05). At a concentration of 100 μg/mL ARP, the activities of these enzymes were restored to 72.88%, 62.81%, and 84.64% of the control group levels, respectively. Furthermore, MDA, a byproduct of lipid peroxidation, serves as a biomarker for oxidative stress and can be utilized to assess the extent of oxidative damage (Xie et al. 2021). As illustrated in Figure 6D, the level of MDA was significantly elevated (*p* < 0.001) in UVB‐treated cells, indicating that HaCaT cells experienced damage. The addition of ARP reduced MDA levels to 4.29% times that of the control group, suggesting the ability of ARP to mitigate oxidative stress‐induced damage and lipid peroxidation.

### 3.11 Effect of ARP on Intracellular ROS in HaCaT Cells

ROS is a collective term for various free radicals, including hydroxyl radicals, superoxide anions, and singlet oxygen, and is commonly used to assess oxidative stress levels (Truong et al. 2020). Figure 6E illustrates the ROS fluorescence images under varying conditions, revealing that UVB‐induced cells exhibit a higher fluorescence intensity, which gradually decreases with the addition of ARP. To more intuitively demonstrate the protective effect of ARP on oxidative‐damaged cells, we quantified the ROS fluorescence intensity under different conditions (Figure 6F). Notably, the ROS level in the damage group was 20.43 times that of the control group, confirming successful model induction. When ARP was added at a concentration of 100 μg/mL, the fluorescence intensity decreased by 25.38% compared to the UVB injury group, approaching levels similar to the control group. This suggests that ARP supplementation can reduce ROS levels in UVB‐induced HaCaT cells, with the effect being dependent on concentration. These findings highlight the potential of ARP as a ROS scavenger.

### 3.12 Identification of Peptide Sequences in ARP

A total of 11,569 peptide sequences were identified from ARP using LC–MS/MS. As predicted by Peptide Ranker, 5088 peptides were identified as potential bioactive peptides (Figure S1A,B). Specifically, these peptides had PeptideRanker scores greater than 0.5, representing 43.98% of all identified peptide sequences. Figure S1C illustrates that there are 40 peptides with PeptideRanker scores exceeding 0.95, which accounts for 0.35% of the total peptides. The biological activities of these 40 polypeptides were predicted using BIOEPE‐UMW, with the results presented in Table S4. Notably, 21 out of the 40 peptides demonstrated potential antioxidant activity (see Figure S1D).

### 3.13 Antioxidant Peptide Property Prediction

**FIGURE 7:**
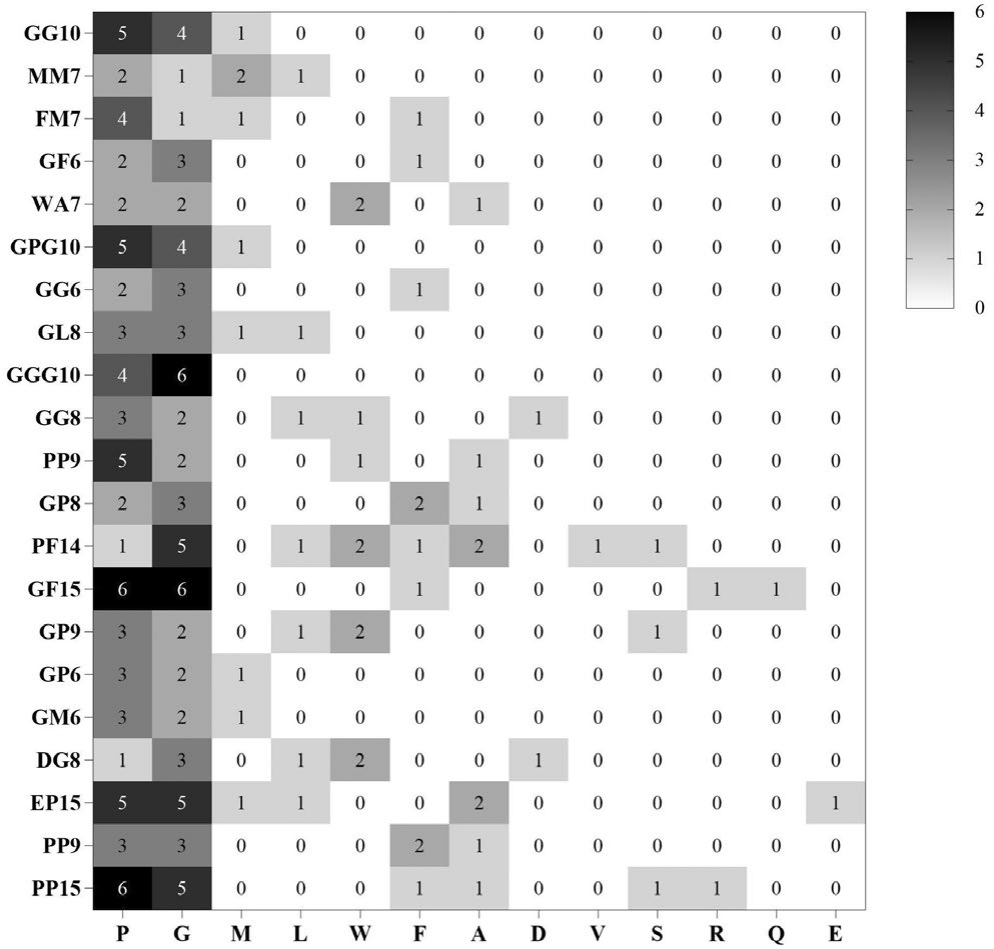
Analysis of the amino acid composition of the screened antioxidant peptides.

The properties of the 21 identified antioxidant peptides were predicted, with the results presented in Table 4. These peptides consist of 6–15 amino acids and possess a molecular weight ranging from 500 to 1400 Da, which aligns with the typical characteristics of antioxidant peptides (Cao et al. 2022). Toxicity predictions indicated that only GG10, GL8, and QF15 were classified as toxic. Research has demonstrated that when the GRAVY value is greater than 0, the peptide is hydrophilic and exhibits increased solubility in water; conversely, when GRAVY is less than 0 (Fu et al. 2019; Nai et al. 2022), the peptide is more hydrophobic and exhibits lower solubility. Thus, GRAVY can be utilized to assess the solubility of peptides. The prediction results reveal that, with the exception of MM7, GP8, PF14, and PGP9, the remaining peptides demonstrate good solubility. Cell membrane permeability is crucial for the biological activity of peptides, and CPPpred can predict the potential of peptides to penetrate cell membranes (Vishnepolsky et al. 2019). A CPPpred value closer to 1 indicates a higher likelihood of cell penetration, while a value closer to 0 suggests a lower likelihood. Meng et al. (2025) established a threshold of CPPpred > 0.1 for peptide screening. Following relevant standards, we identified that only GM6, DG8, EP15, PGP9, and PP15 had CPPpred values below 0.1, signifying that the other 16 peptides possess strong cell penetration capabilities. Additionally, protease degradation is a critical factor that can lead to the reduction or loss of biological activity in functional peptides during use. Therefore, we assessed whether the 21 screened peptides contained trypsin, pepsin, or chymotrypsin digestive sites. Notably, GG10, FM7, GPG10, GGG10, PP9, GP6, and GM6 were found to lack enzyme cleavage sites for these three digestive enzymes. Based on the above prediction results, it is evident that FM7, GPG10, GGG10, PP9, and GP6 exhibit advantages such as nontoxicity, good solubility, effective cell penetration, and the absence of enzymatic hydrolysis sites. The analysis of the amino acid composition of the selected antioxidant peptides revealed that peptides are primarily composed of glycine and proline (Figure 7), consistent with the amino acid composition analysis of ARP presented in Section 3.6. Therefore, it is speculated that this observation may be attributed to the amino acid composition of the source proteins.

**TABLE 4:** Antioxidant peptide property prediction.

No.	Name	Peptide sequence	Amino acid	Mw	PeptideRanker score	Toxin	GRAVY	CPPpred	Trypsin/pepsin/chymotrypsin digestive site
1	GG10	GPPGPPGPMG	10	863.007	0.975508	Yes	−0.77	0.119417	0/0/0
2	MM7	MGWPLPM	7	831.072	0.975364	No	0.44	0.237492	0/1/0
3	FM7	FPPGPPM	7	741.911	0.97492	No	−0.3	0.221604	0/0/0
4	GF6	GPPGGF	6	530.582	0.972814	No	−0.27	0.187067	0/1/0
5	WA7	WGPWPGA	7	769.858	0.972659	No	−0.57	0.183597	0/0/1
6	GPG10	GPPGPPGMPG	10	863.007	0.965658	No	−0.77	0.176453	0/0/0
7	GG6	GPPGFG	6	530.582	0.962685	No	−0.27	0.172385	0/1/1
8	GL8	GPPGPMGL	8	724.881	0.960652	Yes	−0.04	0.168173	0/1/0
9	GGG10	GGGPPPPGGG	10	748.795	0.959546	No	−0.88	0.148413	0/0/0
10	GG8	GPLPDPWG	8	837.932	0.959412	No	−0.78	0.144649	0/1/1
11	PP9	PGPWPPGAP	9	874.996	0.958949	No	−0.88	0.140174	0/0/0
12	GP8	GPAGGFFP	8	748.838	0.958891	No	0.38	0.133342	0/2/1
13	PF14	PGAGWVGGSLGWAF	14	1361.524	0.95768	No	0.59	0.120241	0/7/2
14	QF15	QGPGGPPGRPGPPGF	15	1374.525	0.95695	Yes	−1.15	0.118902	0/0/0
15	GP9	GPLWWPSGP	9	996.134	0.956905	No	−0.49	0.111002	0/2/1
16	GP6	GPPGMP	6	554.669	0.956143	No	−0.62	0.110043	0/0/0
17	GM6	GPPGPM	6	554.669	0.955937	No	−0.62	0.0719604	0/0/0
18	DG8	DLWGPGWG	8	886.963	0.954746	No	−0.54	0.0647246	0/3/2
19	EP15	EGPPGPAGPAGLMGP	15	1304.493	0.95437	No	−0.28	0.0599388	0/2/0
20	PGP9	PGAFFGPGP	9	845.955	0.953082	No	0.16	0.0582664	0/2/2
21	PP15	PGPSGPPGPRGFAGP	15	1347.499	0.951861	No	−0.82	0.0581282	1/1/1

### 3.14 Molecular Docking of ARP and DPPH/ABTS

**FIGURE 8:**
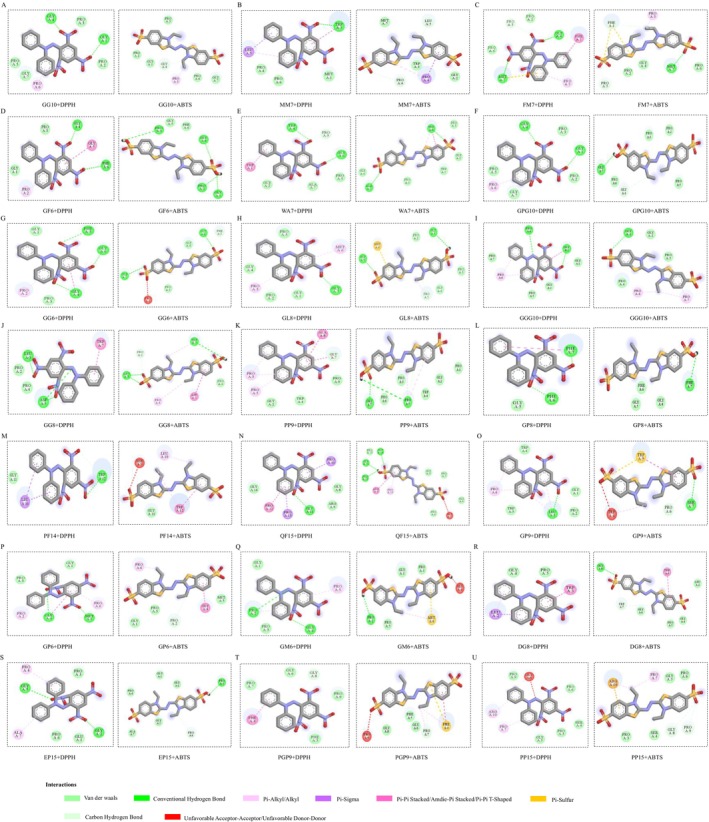
Docking results of 21 antioxidant peptides with DPPH and ABTS. The left side of each figure presents the two‐dimensional correlation diagram of docking with DPPH, while the right side displays the two‐dimensional correlation diagram of docking with ABTS.

DPPH and ABTS are two stable free radicals that are easily detectable and reproducible, making them commonly used for evaluating the free radical scavenging ability of antioxidants, including antioxidant peptides. Consequently, 21 antioxidant peptides were subjected to molecular docking with DPPH and ABTS to analyze their binding abilities and mechanisms of action with these free radicals. The two‐dimensional molecular interaction diagrams and results of the antioxidant peptide docking are presented in Table 5 and Figure 8. The binding energies of the 21 antioxidant peptides to DPPH ranged from −3.3 to −4.6 kcal/mol, while the binding energies to ABTS varied from −3.1 to −4.5 kcal/mol. A docking binding energy of less than 0 indicates that the ligand and receptor can spontaneously combine. Thus, all antioxidant peptides are capable of spontaneously binding with DPPH and ABTS. Further analysis of the binding sites between the free radicals and antioxidant peptides revealed that the binding sites of the antioxidant peptides include Gly, Pro, Trp, Leu, Phe, Met, Ala, Asp, Arg, and Ser. Among these, Gly and Pro exhibited the highest frequency and are most likely to form hydrogen bonds and hydrophobic interactions with DPPH and ABTS, playing a crucial role in the antioxidant effects of the peptides. Finally, the antioxidants screened in Section 3.13 were analyzed and ranked based on their binding abilities to DPPH. From highest to lowest binding ability, the ranking is as follows: FM7 (−4.6 kcal/mol) > PP9 (−3.9 kcal/mol) > GPG10 (−3.7 kcal/mol) ≥ GP6 (−3.7 kcal/mol) > GGG10 (−3.6 kcal/mol). When ranked according to binding ability to ABTS, the order is: FM7 (−4.2 kcal/mol) > PP9 (−3.8 kcal/mol) > GPG10 (−3.6 kcal/mol) > GP6 (−3.4 kcal/mol) > GGG10 (−3.2 kcal/mol). Therefore, FM7, PP9, and GPG10, which demonstrate strong binding abilities, were selected for further research.

**TABLE 5:** Analysis of docking results of antioxidant peptides with DPPH and ABTS.

Peptide	DPPH				ABTS			
	Binding energy (kcal/mol)	Number of classical H‐bonds	Number of hydrophobic interactions	Amino acid residues	Binding energy (kcal/mol)	Number of classical H‐bonds	Number of hydrophobic interactions	Amino acid residues
GG10	−3.6	2	1	Gly1, Gly4, Pro6	−3.6	0	1	Pro3
MM7	−4.5	1	3	Trp3, Leu5	−4.5	0	2	Pro4
FM7	−4.6	2	2	Phe1, Gly4, Pro5, Met7	−4.2	1	1	Pro3, Met7
GF6	−3.4	2	2	Pro2, Gly4, Gly5, Phe6	−3.0	4	0	Gly1, Pro2, Pro3, Gly4
WA7	−3.6	2	1	Trp1, Trp4, Gly6	−3.9	2	2	Trp4, Ala7
GPG10	−3.7	2	1	Gly1, Gly4, Pro6	−3.6	1	0	Gly7
GG6	−3.8	3	2	Pro2, Gly4, Phe5, Gly6	−3.1	2	0	Gly1, Gly6
GL8	−3.3	1	2	Pro3, Met6, Gly7	−3.6	2	1	Gly1, Pro5, Gly7
GGG10	−3.6	2	3	Gly2, Pro4, Pro6	−3.4	1	3	Gly3, Pro6, Pro7
GG8	−4.2	3	1	Leu3, Asp5, Trp7	−4.0	3	3	Leu3, Pro4, Asp5, Trp7
PP9	−3.9	0	5	Pro3, Pro5, Gly7, Ala8	−3.8	2	2	Pro5, Gly7
GP8	−3.6	2	1	Phe6, Phe7	−3.2	0	1	Phe7
PF14	−3.8	1	2	Leu10, Trp12	−4.5	0	3	Leu10, Trp12
QF15	−3.6	1	3	Pro10, Gly11, Pro12, Pro13	−4.1	3	2	Arg9, Pro10, Gly11, Pro12, Gly14
GP9	−4.2	1	4	Leu3, Pro6	−4.0	1	2	Trp4, Trp5, Ser7
GP6	−3.7	2	3	Pro2, Gly4, Met5, Pro6	−3.2	0	2	Gly4, Pro6
GM6	−3.3	2	2	Pro, Gly4, Pro5	−3.3	1	1	Pro2, Met6
DG8	−3.7	0	3	Leu2, Trp3	−3.8	1	1	Trp3, Gly8
EP15	−3.7	2	2	Gly2, Pro4, Gly5, Ala7	−3.4	1	0	Pro3
PGP9	−3.6	0	1	Phe4	−3.6	0	3	Phe4, Pro7
PP15	−4.1	0	2	Pro7, Arg10	−3.9	0	2	Pro7, Arg9

### 3.15 Molecular Docking of ARP and Keap1

**FIGURE 9:**
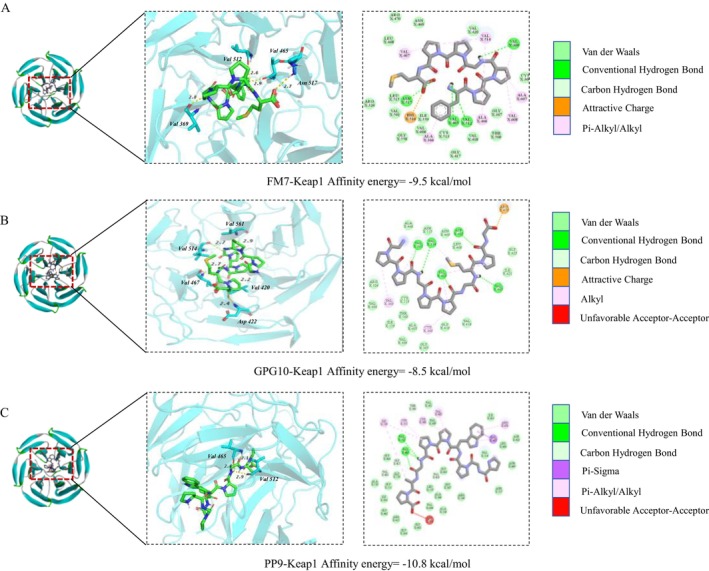
Docking results of FK7, PP9, and GPG10 with Keap1. (A) FK7; (B) GPG10; (C) PP9.

Within the body, multiple signaling pathways collaborate to maintain redox homeostasis. Among these, the Nrf2/Keap1 signaling pathway plays a crucial role in sustaining cellular homeostasis and responding to oxidative stress (Lv et al. 2017). Consequently, we conducted molecular docking studies of FK7, PP9, GPG10, GGG10, and GP6 with Keap1, with the docking results presented in Table S5. Liu et al. suggest that a docking binding energy of less than −7 kcal/mol indicates a strong binding affinity between the ligand and the receptor. Thus, these five antioxidant peptides exhibit a strong binding capability to Keap1. Notably, FK7 (−9.5 kcal/mol), PP9 (−10.8 kcal/mol), and GPG10 (−8.5 kcal/mol) demonstrated the most favorable docking interactions, which align with the screening results from DPPH and ABTS assays. Visual analysis of the docking results for FK7, PP9, and GPG10 with Keap1 is illustrated in Figure 9. Conventional hydrogen bonds and hydrophobic interactions represent the two predominant forces in molecular docking; therefore, we conducted an analysis and statistical evaluation of these interactions. FK7 can form four hydrogen bonds with Val369, Val465, Val512, and Asn517 of Keap1, while also engaging in hydrophobic interactions with Ala366, Val369, Ala466, Val467, Val514, Ala607, and Val608. PP9 forms three hydrogen bonds with Val465 and Val512 of Keap1 and exhibits hydrophobic interactions with Cys368, Val420, Val467, Arg470, Cys513, and Ile559. GPG10 establishes five hydrogen bonds with Val420, Asp422, Val467, Val514, and Val561 of Keap1, along with hydrophobic interactions with Cys368, Val369, Val420, Val514, and Val561. Notably, the Val residues of Keap1 are likely to interact with these three antioxidant peptides. These findings suggest that FK7, PP9, and GPG10 may influence the interaction between Keap1 and Nrf2 by binding to Keap1, potentially leading to the dissociation and release of Nrf2, which could subsequently affect the expression of downstream genes and oxidative stress‐induced damage. In future experiments, combining qPCR and Western Blot could further verify the interaction mechanism between the peptides and the Keap1/Nrf2 pathway, thus enhancing the clarification of the antioxidant peptides' action mechanism.

## 4 Conclusion

This study optimized the extraction conditions of antioxidant peptides from ARP using single‐factor and response surface methodologies. The analysis revealed that ARP consists of a mixture of antioxidant peptides that are rich in glycine and proline, exhibiting high biosafety and primarily composed of small molecular peptides. In the UVB‐induced oxidative damage model, ARP provided cellular protection by enhancing the activity of antioxidant enzymes and reducing the levels of MDA and ROS accumulation. Peptidomics combined with machine learning identified 21 antioxidant peptides, among which FK7, PP9, and GPG10 exhibited non‐toxicity, good solubility, effective cell permeability, and a lack of digestion sites for pepsin, trypsin, and chymotrypsin. Docking studies with DPPH, ABTS, and Keap1 further elucidated the binding sites and mechanisms of action of these antioxidant peptides.

## Author Contributions

**Xingyu Xiao:** conceptualization (lead), data curation (supporting), formal analysis (lead), investigation (lead), methodology (lead), writing – original draft (lead). **Xi Chen:** conceptualization (lead), data curation (lead), formal analysis (supporting), investigation (supporting), methodology (supporting). **Libo Zhang:** data curation (supporting), investigation (supporting), methodology (supporting), writing – original draft (supporting). **Yi Li:** data curation (lead), funding acquisition (supporting), investigation (supporting), methodology (supporting). **Qinchuan Lv:** investigation (supporting), methodology (supporting). **Tong Su:** investigation (supporting), methodology (supporting). **Jiayuan Fang:** formal analysis (supporting), investigation (supporting). **Shuo Zheng:** formal analysis (supporting), investigation (supporting). **Xunming Zhang:** data curation (supporting), investigation (supporting), software (supporting). **Linlin Hao:** project administration (lead), supervision (supporting), writing – review and editing (lead). **Shuqin Cheng:** funding acquisition (lead), project administration (supporting), writing – review and editing (supporting).

## Conflicts of Interest

The authors declare no conflicts of interest.

## Supporting information


**Data S1:** fsn370878‐sup‐0001‐Supinfo1.docx.

## Data Availability

The data of this study are available from the corresponding author upon reasonable request.
