# Supplementary material for: Identification and Characterization of Glycine‐ and Proline‐Rich Antioxidant Peptides From Antler Residues Based on Peptidomics, Machine Learning, and Molecular Docking
Source: Food Sci Nutr. 2025 Sep 1;13(9):e70878. doi: 10.1002/fsn3.70878 (PMC12400163; doi:10.1002/fsn3.70878)
Supplement: Supplementary file 1 — Data S1: fsn370878‐sup‐0001‐Supinfo1.docx. [file FSN3-13-e70878-s001.docx]

**Supplementary Material**

**Identification and characterization of glycine- and proline-rich antioxidant peptides from antler residues based on peptidomics, machine learning and molecular docking**

Xingyu Xiao^a,1^, Xi Chen^a,1^, Libo Zhang^a^, Yi Li^a^, Qinchuan Lv^a^, Tong Su^a^, Jiayuan Fang^a^, Shuo Zheng^a^, Xunming Zhang^a^, Linlin Hao*^a^, Shuqin Cheng*^b,c^

Xingyu Xiao^1^: [xyxiao23@mails.jlu.edu.cn](mailto:xyxiao23@mails.jlu.edu.cn)

Xi Chen^1^: xic21@mails.jlu.edu.cn

Libo Zhang: [zlb22@mails.jlu.edu.cn](mailto:zlb22@mails.jlu.edu.cn)

Yi Li: [yil22@mails.jlu.edu.cn](mailto:yil22@mails.jlu.edu.cn)

Qinchuan Lv: lvqc9921@mails.jlu.edu.cn

Tong Su: sutong9921@mails.jlu.edu.cn

Jiayuan Fang: fangjy23@mails.jlu.edu.cn

Shuo Zheng: zhengshuo22@mails.jlu.edu.cn

Xunming Zhang: [xunming22@mails.jlu.edu.cn](mailto:xunming22@mails.jlu.edu.cn)

Linlin Hao*: [haolinlin@jlu.edu.cn](mailto:haolinlin@jlu.edu.cn)

Shuqin Cheng*: chengsq19@mails.jlu.edu.cn

^a^College of Animal Science, Jilin University, Changchun, 130062, China.

^b^College of Veterinary Medicine,Jilin University, Changchun, 130062, China.

^c^Handan Vocational College of Science and Technology, Handan, 056004, China

^1^ These authors contributed equally to this work

^*^Corresponding author

Supplement table 1 The factor levels of response surface experiment

| Level | Factors | | |
| --- | --- | --- | --- |
|  | A enzyme concentration （U/g） | C Time（h） | C pH |
| -1 | 1000 | 7 | 6.0 |
| 0 | 2000 | 8 | 6.5 |
| 1 | 3000 | 9 | 7.0 |

Supplement table 2 Response surface experimental design and results.

| Number | A Enzyme concentration（U/g） | B Time (h) | C pH | DPPH (%) | DH (%) |
| --- | --- | --- | --- | --- | --- |
| 1 | -1（1000） | 0（8） | -1（6） | 61.01 | 10.05 |
| 2 | 0（2000） | -1（7） | 1（7） | 62.83 | 11.53 |
| 3 | 1（3000） | -1（7） | 0（6.5） | 61.01 | 12.51 |
| 4 | 0（2000） | 0（8） | 0（6.5） | 72.12 | 15.86 |
| 5 | 0（2000） | 0（8） | 0（6.5） | 70.28 | 15.75 |
| 6 | -1（1000） | -1（7） | 0（6.5） | 64.91 | 10.79 |
| 7 | 0（2000） | 0（8） | 0（6.5） | 71.97 | 15.69 |
| 8 | 0（2000） | 1（9） | -1（6） | 65.75 | 9.36 |
| 9 | 0（2000） | -1（7） | -1（6） | 62.49 | 11.10 |
| 10 | -1（1000） | 1（9） | 0（6.5） | 54.90 | 9.43 |
| 11 | 0（2000） | 1（9） | 1（7） | 47.21 | 11.28 |
| 12 | 0（2000） | 0（8） | 0（6.5） | 71.66 | 16.01 |
| 13 | 1（3000） | 0（8） | 1（7） | 53.42 | 9.68 |
| 14 | -1（1000） | 0（8） | 1（7） | 53.95 | 11.79 |
| 15 | 1（3000） | 0（8） | -1（6） | 58.48 | 12.02 |
| 16 | 1（3000） | 1（9） | 0（6.5） | 50.26 | 9.49 |
| 17 | 0（2000） | 0（8） | 0（6.5） | 66.28 | 15.51 |

Supplement table 3 Statistics of relative molecular masse distribution

| Molecular weight ranges | Percentage |
| --- | --- |
| >10kDa | 0.83 |
| 5-10kDa | 0.19 |
| 3-5kDa | 0.45 |
| 1-3kDa | 4.93 |
| <1kDa | 93.6 |

Table 4 BIOPEP-UWM biological activity prediction.

| **No.** | | **Sequence** | | **ao** | | **ah** | | **am** | | **inh** | | **re** | | **pam** | | **glui** | | **dpp** | | **che** | | **at** | | **ren** | | **ace2** | | **xaap** | | **lcp** | | **tpp2** | | **dpp3** | | **acylp** | | **caa** | | **acar** | | **op** | | **neul** | | **ne** | | **preud** | | **hur** | | **ac** | | **leut** | | **im** | | **bin** | | **apr** | |
| --- | --- | --- | --- | --- | --- | --- | --- | --- | --- | --- | --- | --- | --- | --- | --- | --- | --- | --- | --- | --- | --- | --- | --- | --- | --- | --- | --- | --- | --- | --- | --- | --- | --- | --- | --- | --- | --- | --- | --- | --- | --- | --- | --- | --- | --- | --- | --- | --- | --- | --- | --- | --- | --- | --- | --- | --- | --- | --- | --- | --- | --- |
| 1 | | GPPGPPGPMG | | + | | + | | + | | + | | + | | + | | + | | + | | + | | + | |  | |  | |  | |  | |  | |  | |  | |  | |  | |  | |  | |  | |  | |  | |  | |  | |  | |  | |  | |
| 2 | | MGPSFPF | |  | | + | | + | |  | | + | |  | |  | | + | |  | | + | | + | | + | |  | |  | |  | |  | |  | |  | |  | |  | |  | |  | |  | |  | |  | |  | |  | |  | |  | |
| 3 | | MGWPLPM | | + | | + | |  | |  | |  | |  | |  | | + | |  | |  | |  | |  | | + | | + | | + | |  | |  | |  | |  | |  | |  | |  | |  | |  | |  | |  | |  | |  | |  | |
| 4 | | FPPGPPM | | + | | + | | + | | + | | + | | + | | + | | + | | + | | + | |  | |  | |  | |  | |  | |  | |  | |  | |  | |  | |  | |  | |  | |  | |  | |  | |  | |  | |  | |
| 5 | | GPPGGF | | + | | + | | + | |  | | + | | + | | + | | + | |  | | + | |  | |  | |  | |  | | + | | + | | + | | + | |  | |  | |  | |  | |  | |  | |  | |  | |  | |  | |  | |
| 6 | | WGPWPGA | | + | | + | | + | |  | | + | | + | |  | | + | |  | | + | | + | |  | |  | |  | |  | |  | |  | |  | | + | |  | |  | |  | |  | |  | |  | |  | |  | |  | |  | |
| 7 | | FPPPLG | |  | | + | |  | |  | |  | |  | | + | | + | |  | |  | |  | |  | | + | | + | |  | |  | |  | |  | |  | | + | |  | |  | |  | |  | |  | |  | |  | |  | |  | |
| 8 | | GIPMPPPPMGLP | |  | | + | |  | |  | |  | |  | | + | | + | |  | |  | |  | |  | |  | | + | |  | |  | |  | |  | |  | |  | |  | |  | |  | |  | |  | |  | |  | |  | |  | |
| 9 | | GPGPGF | |  | | + | | + | | + | | + | | + | |  | | + | | + | | + | |  | |  | |  | |  | | + | |  | | + | |  | |  | |  | |  | |  | |  | |  | |  | |  | |  | |  | |  | |
| 10 | | LPGPFL | |  | | + | | + | | + | | + | | + | |  | | + | | + | | + | |  | | + | |  | | + | |  | | + | |  | |  | |  | |  | |  | |  | |  | |  | |  | |  | |  | |  | |  | |
| 11 | | GPSPGPF | |  | | + | | + | | + | | + | | + | |  | | + | | + | | + | |  | |  | | + | |  | |  | | + | |  | |  | |  | |  | |  | |  | |  | |  | |  | |  | |  | |  | |  | |
| 12 | | GPPGPPGMPG | | + | | + | | + | | + | | + | | + | | + | | + | | + | | + | |  | |  | |  | |  | |  | |  | |  | |  | |  | |  | |  | |  | |  | |  | |  | |  | |  | |  | |  | |
| 13 | | GPPGFG | | + | | + | | + | |  | | + | | + | | + | | + | |  | | + | |  | |  | |  | |  | | + | | + | | + | |  | |  | |  | | + | |  | |  | |  | |  | |  | |  | |  | |  | |
| 14 | | GPAGFPPLMH | |  | | + | | + | |  | | + | |  | | + | | + | |  | | + | |  | |  | | + | | + | | + | | + | | + | |  | |  | |  | |  | | + | |  | |  | |  | |  | |  | |  | |  | |
| 15 | | GPPGPMGL | | + | | + | | + | | + | | + | | + | | + | | + | | + | | + | |  | |  | |  | |  | |  | |  | |  | |  | |  | |  | |  | |  | |  | |  | |  | |  | |  | |  | |  | |
| 16 | | GGGPPPPGGG | | + | | + | | + | | + | | + | | + | | + | | + | |  | | + | |  | |  | |  | |  | |  | |  | |  | |  | |  | |  | |  | |  | |  | |  | |  | |  | |  | |  | |  | |
| 17 | | GPLPDPWG | | + | | + | | + | |  | | + | |  | |  | | + | |  | | + | | + | |  | | + | | + | |  | |  | |  | |  | |  | |  | |  | |  | |  | |  | |  | |  | |  | |  | |  | |
| 18 | GPLPDPWG | | + | | + | | + | |  | | + | |  | |  | | + | |  | | + | | + | |  | | + | | + | |  | |  | |  | |  | |  | |  | |  | |  | |  | |  | |  | |  | |  | |  | |  | |  |
| 19 | PGPWPPGAP | | + | | + | | + | | + | | + | | + | | + | | + | | + | | + | |  | |  | |  | |  | | + | |  | |  | |  | | + | |  | |  | |  | |  | |  | |  | |  | |  | |  | |  | |  |
| 20 | GPAGGFFP | | + | | + | | + | |  | | + | |  | |  | | + | |  | | + | |  | |  | |  | |  | | + | | + | | + | | + | |  | |  | |  | |  | | + | |  | |  | |  | |  | |  | |  | |  |
| 21 | GPAGPF | |  | | + | | + | |  | | + | |  | |  | | + | |  | | + | |  | | + | |  | |  | |  | | + | |  | |  | |  | |  | |  | |  | |  | |  | |  | |  | |  | |  | |  | |  |
| 22 | PGAGWVGGSLGWAF | | + | | + | | + | |  | | + | | + | |  | | + | |  | | + | |  | |  | |  | |  | | + | |  | |  | |  | | + | |  | |  | |  | |  | | + | |  | |  | |  | |  | | + | |  |
| 23 | GPGGGPGSCGGPGVGGGPGG | |  | | + | | + | |  | | + | | + | |  | | + | |  | | + | |  | |  | |  | |  | |  | |  | |  | |  | |  | |  | |  | |  | |  | |  | | + | |  | |  | |  | |  | |  |
| 24 | QGPGGPPGRPGPPGF | | + | | + | | + | | + | | + | | + | | + | | + | | + | | + | |  | |  | |  | |  | | + | | + | | + | |  | |  | |  | | + | | + | |  | |  | |  | |  | |  | |  | |  | |  |
| 25 | GPLWWPSGP | | + | | + | | + | |  | | + | |  | |  | | + | |  | | + | | + | |  | | + | | + | |  | | + | |  | |  | |  | |  | |  | |  | |  | | + | |  | |  | |  | |  | |  | |  |
| 26 | GPGFPG | |  | | + | | + | |  | | + | | + | |  | | + | |  | | + | |  | |  | |  | |  | | + | | + | | + | |  | |  | |  | |  | |  | |  | |  | |  | |  | |  | |  | |  | |  |
| 27 | GPPGMP | | + | | + | | + | |  | | + | | + | | + | | + | |  | | + | |  | |  | |  | |  | |  | |  | |  | |  | |  | |  | |  | |  | |  | |  | |  | |  | |  | |  | |  | |  |
| 28 | GPPGPM | | + | | + | | + | | + | | + | | + | | + | | + | | + | | + | |  | |  | |  | |  | |  | |  | |  | |  | |  | |  | |  | |  | |  | |  | |  | |  | |  | |  | |  | |  |
| 29 | PGSPGPF | |  | | + | | + | | + | | + | | + | |  | | + | | + | | + | |  | | + | |  | |  | |  | | + | |  | |  | |  | |  | |  | |  | |  | |  | |  | |  | |  | |  | |  | |  |
| 30 | DLWGPGWG | | + | | + | | + | |  | | + | | + | |  | | + | |  | | + | | + | |  | |  | |  | | + | | + | |  | |  | |  | |  | |  | |  | |  | | + | |  | |  | |  | |  | |  | |  |
| 31 | EGPPGPAGPAGLMGP | | + | | + | | + | | + | | + | | + | | + | | + | | + | | + | |  | |  | |  | |  | |  | |  | |  | |  | |  | |  | |  | | + | |  | |  | |  | |  | |  | | + | |  | |  |
| 32 | GPGPGPAFGPGPA | |  | | + | | + | | + | | + | | + | |  | | + | | + | | + | |  | |  | |  | |  | | + | |  | |  | |  | |  | |  | | + | |  | |  | |  | |  | |  | |  | |  | |  | |  |
| 33 | GPGPMGLM | |  | | + | | + | | + | | + | | + | |  | | + | | + | | + | |  | |  | |  | |  | |  | |  | |  | |  | |  | |  | |  | |  | |  | |  | |  | |  | |  | |  | |  | |  |
| 34 | PGPGPGPAFGPGPA | |  | | + | | + | | + | | + | | + | |  | | + | | + | | + | |  | |  | |  | |  | | + | |  | |  | |  | |  | |  | | + | |  | |  | |  | |  | |  | |  | |  | |  | |  |
| 35 | IPGPLF | |  | | + | | + | | + | | + | | + | |  | | + | | + | | + | |  | |  | | + | | + | |  | |  | |  | |  | |  | |  | |  | |  | |  | |  | |  | |  | |  | |  | |  | |  |
| 36 | PGAFFGPGP | | + | | + | | + | | + | | + | | + | |  | | + | | + | | + | |  | |  | |  | |  | | + | |  | |  | |  | | + | |  | | + | |  | | + | |  | |  | |  | |  | |  | |  | |  |
| 37 | GPGSPPF | |  | | + | | + | |  | | + | | + | | + | | + | |  | | + | |  | | + | |  | |  | |  | | + | |  | |  | |  | |  | |  | |  | |  | |  | |  | |  | |  | |  | |  | |  |
| 38 | PGPSGPPGPRGFAGP | | + | | + | | + | | + | | + | | + | | + | | + | | + | | + | |  | |  | |  | |  | | + | | + | | + | |  | | + | |  | |  | |  | |  | |  | |  | | + | |  | |  | |  | |  |
| 39 | GPAGFL | |  | | + | | + | |  | | + | |  | |  | | + | |  | | + | |  | |  | |  | |  | | + | | + | | + | |  | |  | |  | |  | |  | |  | |  | |  | |  | | + | |  | | + | |  |
| 40 | FPPPGI | |  | | + | | + | | + | | + | | + | | + | | + | |  | | + | |  | |  | |  | |  | |  | |  | |  | |  | |  | |  | |  | |  | |  | |  | |  | |  | |  | |  | |  | |  |

**am:** antiamnestic; **ah:** ACE inhibitor; **ao:** antioxidative; **re:** regulating; **inh:** inhibitor; **at:** antithrombotic；**che:** chemotactic; **pam:** PAM inhibitor; **glui:** alpha-glucosidase inhibitor; **dpp:** dipeptidyl peptidase IV inhibitor; **ace2:** ACE2 inhibitor; **ren:** renin inhibitor; **xaap:** xaa-pro inhibitor; **lcp:** lactocepin inhibitor; **dpp3:** dipeptidyl peptidase III inhibitor; **tpp2:** inhibitor of tripeptidyl peptidase II; **acylp:** acylaminoacyl peptidase inhibitor; **caa:** inhibitor of cytosol alanyl aminopeptidase; **acar:** alanine carboxypeptidase inhibitor; **op:**opioid; neul: neprilysin inhibitor; **ne:**neuropeptide; **preud:** pseudolysin inhibitor：**hur:** hypouricemic; **ac:** anticancer; **leut:** leucyltransferase inhibitor; **im:** immunostimulating; **bin:** binding; **apr:** activating ubiquitin-mediated proteolysis

Supplement table 5 Analysis of docking results of FK7, PP9, and GPG10 with Keap1

| **Peptide** | **Binding energy (kcal/mol)** | **Number of classical H-bonds** | **Number of**  **Hydrophobic interactions** | **Amino acid residues** |
| --- | --- | --- | --- | --- |
| FM7 | -9.5 | 4 | 8 | Ala366、Val369、Val465、Ala466、Val467、Val514、Val512、Asn517、Ala607、Val608 |
| GGG10 | -7.2 | 6 | 7 | Arg326、Val369、Gly371、Ala466、Val467、Cys513、Val514、Thr560、Val561、Val608 |
| GP6 | -7.7 | 3 | 4 | Val369、Ala466、Val467、Cys513、Val561、Ala607 |
| GPG10 | -8.5 | 5 | 6 | Cys368、Val369、Val420、Asp422、Val467、Val514、Val561 |
| PP9 | -10.8 | 3 | 8 | Cys368、Val420、Val465、Val467、Arg470、Val512、Cys513、Ile559 |


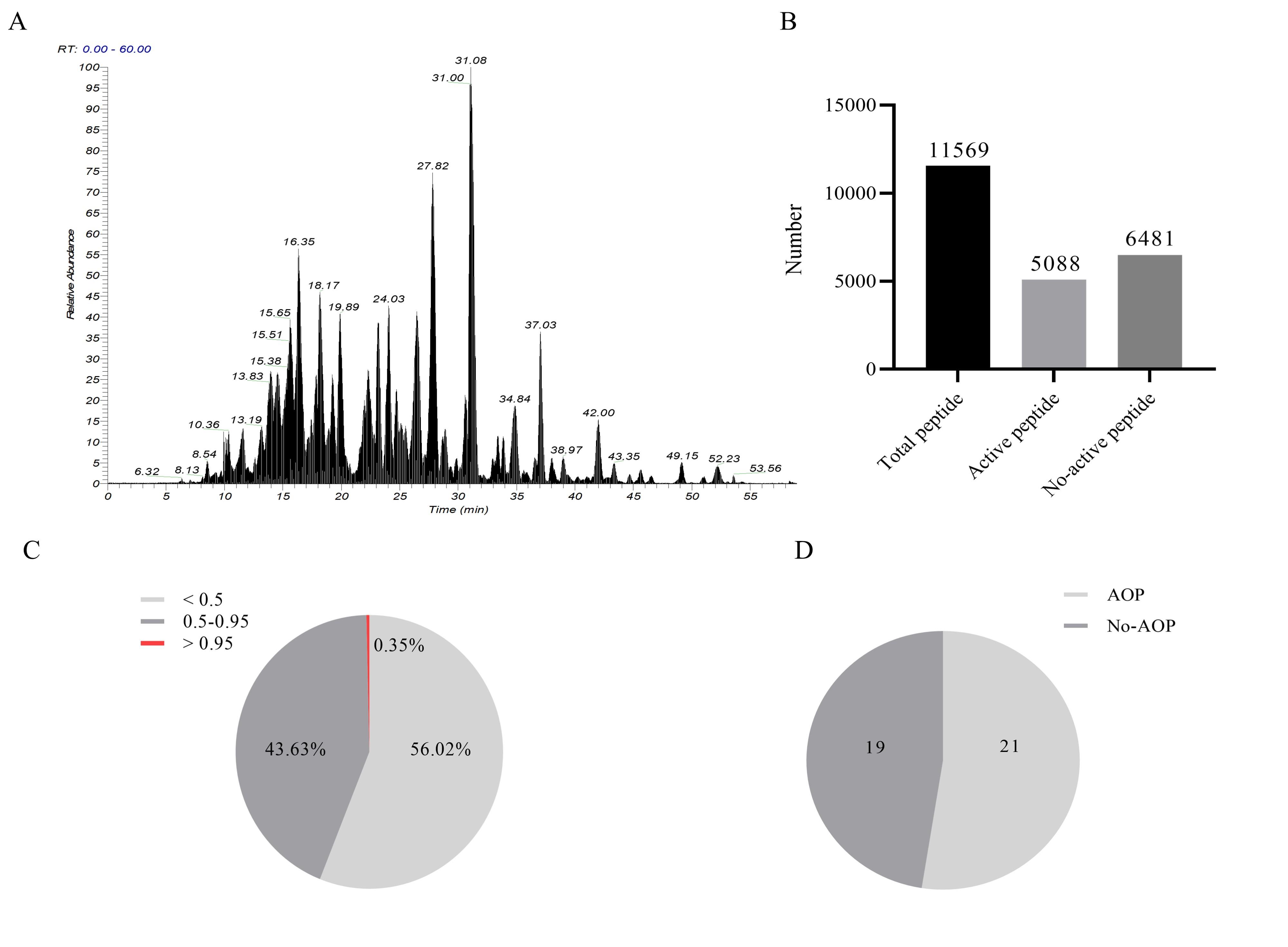


**Supplement figure 1**: A. Mass spectrometry detection results; B. pepitidebank prediction results; C. Bioactive peptide pepitidebank score proportion; D. BIOEPE-UMW prediction results.
